# Bioinformatics and Molecular Insights to Anti-Metastasis Activity of Triethylene Glycol Derivatives

**DOI:** 10.3390/ijms21155463

**Published:** 2020-07-30

**Authors:** Vidhi Malik, Sukant Garg, Sajal Afzal, Jaspreet Kaur Dhanjal, Chae-Ok Yun, Sunil C. Kaul, Durai Sundar, Renu Wadhwa

**Affiliations:** 1 DAILAB, Department of Biochemical Engineering & Biotechnology, Indian Institute of Technology (IIT) Delhi, Hauz Khas, New Delhi 110 016, India; vidhi0205@gmail.com; 2 AIST-INDIA DAILAB, DBT-AIST International Center for Translational & Environmental Research (DAICENTER), National Institute of Advanced Industrial Science & Technology (AIST), Tsukuba 305 8565, Japan; sukant.garg@aist.go.jp (S.G.); sajal.afzal@aist.go.jp (S.A.); jaspreetk.dhanjal@aist.go.jp (J.K.D.); s-kaul@aist.go.jp (S.C.K.); 3 Department of Bioengineering, College of Engineering, Hanyang University, Seoul 133-791, Korea; chaeok@hanyang.ac.kr

**Keywords:** triethylene glycol derivatives, MMP, VEGF, mortalin, inhibitor, ECM enhancer, EMT reversal

## Abstract

The anti-metastatic and anti-angiogenic activities of triethylene glycol derivatives have been reported. In this study, we investigated their molecular mechanism(s) using bioinformatics and experimental tools. By molecular dynamics analysis, we found that (i) triethylene glycol dimethacrylate (TD-10) and tetraethylene glycol dimethacrylate (TD-11) can act as inhibitors of the catalytic domain of matrix metalloproteinases (MMP-2, MMP-7 and MMP-9) by binding to the S1’ pocket of MMP-2 and MMP-9 and the catalytic Zn ion binding site of MMP-7, and that (ii) TD-11 can cause local disruption of the secondary structure of vascular endothelial growth factor A (VEGFA) dimer and exhibit stable interaction at the binding interface of VEGFA receptor R1 complex. Cell-culture-based in vitro experiments showed anti-metastatic phenotypes as seen in migration and invasion assays in cancer cells by both TD-10 and TD-11. Underlying biochemical evidence revealed downregulation of VEGF and MMPs at the protein level; MMP-9 was also downregulated at the transcriptional level. By molecular analyses, we demonstrate that TD-10 and TD-11 target stress chaperone mortalin at the transcription and translational level, yielding decreased expression of vimentin, fibronectin and hnRNP-K, and increase in extracellular matrix (ECM) proteins (collagen IV and E-cadherin) endorsing reversal of epithelial–mesenchymal transition (EMT) signaling.

## 1. Introduction

Cancer, a highly complex disease of cell proliferation, is regulated by multiple genes and its micro-environment. Some of the most commonly attributed mechanisms include (i) activation of proto-oncogenes, (ii) inhibition of tumor suppressors, (iii) attainment of autonomous growth signals and resistance to extra-cellular growth-inhibitory signals, (iv) resistance to apoptosis, (v) telomere maintenance and (vi) stimulation of cell migration and neoangiogenesis [1,2,3,4,5,6]. The latter is the prime feature of advanced tumors and primarily involved in the spread of the disease. Metastasis is the process that connects primary and secondary tumor sites of cancer [7]. Cancer cells at the primary site get triggered by pro-migratory factors such as Wnt/β-catenin, hypoxia or TGF-β and undergo epithelial-to-mesenchymal transformation (EMT); these cells lose surface adhesion proteins (such as E-cadherin, ZO-1 and Laminin) and gain mesenchymal proteins (such as N-cadherin, vimentin and matrix metalloproteinases) [8,9]. They attain weak cell–cell interactions, migrate and intravasate into nearby vasculature, circulate to distant (secondary) sites and subsequently colonize. In their gravest forms, they may start to migrate radially into the surrounding secondary site parenchyma. Some of the hallmark cellular proteins responsible for detachment from the primary site and solid tumor growth at the secondary sites are matrix metalloproteinases (MMPs) and vascular endothelial growth factor (VEGF), respectively.

VEGF, or the vascular endothelial growth factor, is an angiogenic growth factor secreted by cancer cells that interacts with the extracellular domain of VEGF receptor located on the endothelial cells lining nearby blood vessels [10]. This interaction allows dimerization and activation of the receptor (tyrosine kinase type) and intracellular transphosphorylation, eventually resulting in the activation of proteins vital to cell survival, proliferation, focal adhesion, angiogenesis and migration, such as the MMPs [11,12]. A number of VEGF inhibitors (bevacizumab, ramucirumab and rituximab), VEGF-R-fusion proteins (aflibercept) and VEGF-R-TKI-inhibitors (sunitinib, sorafenib, vandetanib, pazopanib, regorafenib and axitinib) are currently in use for the treatment of metastatic cancers [13,14,15]. The VEGF gene is located on human chromosome 6, and the protein is expressed abundantly in almost all cancer and few endothelial cells, in response to stimuli such as hypoxia, hypoglycemia, growth factors/cytokines (EGF, FGF, IGF, TGF-β, activin A, IL6, TNF-α) and oncogenes (p53, NFκB, Wnt, TIMP and FAK) [16]. VEGF, synthesized and secreted by the tumor cells, is known to activate VEGF-R-mediated PI3K/Akt and Erk pathways in endothelial cells that cause relaxation of vascular smooth muscle and inhibition of platelet aggregation. Whereas the inhibition of these mechanisms could lead to poor wound healing, their provocation inevitably causes severe bleeding disorders [17,18,19].

MMPs are the zinc-dependent endopeptidases, known to cleave cell-adhesion proteins in the extracellular matrix [20]. Pathologically, these zymogens are regulated by tissue inhibitors of metalloproteinases (TIMPs) and are over-expressed in malignant cancers to form the basis of EMT. However, an attempt to completely abolish their functions is irrational as many of them are physiologically vital for wound healing and cell–cell interactions. There are 28 identified subtypes of MMPs; MMPs can be categorized into collagenases (MMP-1, -8 and -13), stromelysins (MMP-3 and -10), gelatinases (MMP-2 and -9) and matrilysins (MMP-7 and -26) [21]. MMP-2 is one of the proteins produced directly downstream to VEGF [22]. Structurally, these have four major domains–(i) the zinc-interacting cysteine switch containing pro-peptide responsible to keep the protein in an inactivated state, (ii) the zinc-ion binding motif harboring catalytic domain, (iii) the hinge region responsible for enzyme’s stability and (iv) the hemopexin-like C-terminal domain organized into a four-bladed -propeller structure and responsible for protein interaction and substrate stability. MMP-1, -2, -3, -9, -13 and -14 are known to be involved in the cleavage of chemokine ligands, which upon truncation, do not activate inflammatory receptors thus inhibit inflammation [23]. MMP-7 is known to bind to and inhibit the FAS-cleavage, inhibiting apoptosis and thereby contributing to carcinogenesis [24]. Various MMPs have been known to cleave the extracellular domain of E-cadherin that interacts with β-catenin to anchor the cells to surrounding parenchyma [25]. MMP-based ECM-protein cleavage results in detachment of cells from the surrounding parenchyma essential to allow them to metastasize. MMP-2 knockdown was shown to suppress the proliferative capacity of hepatocellular carcinoma cells in vitro [26]. MMP-7 was shown to promote the proliferation of mouse-derived tongue squamous cell carcinoma [27]. MMP-7 was found to be directly involved in cell survival, proliferation, migration and invasion of the cervical cancer cells, and its expression was suggested as a novel prognostic biomarker [28]. MMP-9 was shown to cleave heparan sulfate and secrete VEGF essential for the angiogenesis and progression of colorectal cancer [29]. Inhibition of MMP-2 and -9 decreases migration and angiogenic potential of metastatic retinoblastoma cell lines showing a clear relation of MMPs to VEGF and TGF-1 [30]. The expression of both was shown to directly relate to lymph node metastasis and the stage of tumor. They were suggested to be the key prognostic biomarkers to draw the severity of breast cancers [31]. MMP-mediated metastatic activities may more distinctly be sorted into primary tumor survival and proliferation (MMP-7 and -14), detachment and ECM invasion (MMP-9, -10 and -15), intravasation (MMP-2, -9 and -14), immune evasion (MMP-1, -2, and -9), extravasation (MMP-2, -9 and -14), survival and proliferation at the secondary site (MMP-1, -2, -3, -7, -9, -13 and -14) and angiogenesis (MMP-1, -2, -7, -9 and -14) [32]. Collectively, the MMPs have been shown to play a vital role in cell proliferation, migration, differentiation, angiogenesis and host defense.

For the purpose of anticancer therapy, various synthetic and natural MMP-inhibitors have been under clinical trials [33,34]. Some of them, such as Batimastat (BB-94), Marimastat (BB-2516), Prinomastat (AG-3340), Tanomastat (BAY12-9566) and MMI 270 B (CGS 27023 A), are associated with severe musculoskeletal side-effects. These effects are present in the form of osteoarthritis and are attributed to the degradation of MMP-1 and -13 [35]. Metastat (COL-3, CMT-3) was shown to be beneficial against nonepithelial-type malignancy and is currently under a phase II trial. Doxycycline, currently, is the only U.S. Food and Drug Administration (FDA)-approved MMP-inhibitor indicated for the treatment of periodontitis. Natural inhibitors include shark cartilage and genistein and are known for their broad-spectrum effects against cancer. Whereas the administration of multitudinously targeting MMP inhibitors is known to result in severe musculoskeletal toxicity [32,36], targeting specific MMPs with mild to moderate inhibition may pave the way for the new and safer drug discovery regimens culminating into maximum therapy and minimum adversities. Bloomston et al. (2002) suggested the suitability of MMPs as the pharmacological targets in the treatment of pancreatic cancer [37]. Jakubowska et al. (2016) showed that MMP-2, MMP-7 and MMP-9 are over-expressed in pancreatic ductal carcinoma and closely related to the tumor morphological features such as induction of inflammation, inhibition of necrosis and angiogenesis, respectively [38]. We had earlier demonstrated that the TEG derivatives, triethylene glycol dimethacrylate (TD-10) and tetraethylene glycol dimethacrylate (TD-11), showed anti-metastatic effects in lung cancer A549 cells via inhibition of canonical Wnt/β-catenin signaling and EMT proteins including MMP-2 and VEGF [39]. These derivatives are the methacryloyl derivatives of triethylene glycol; both molecules are the hydration products of ethylene oxide and are produced when ethylene is oxidized at high temperature in the presence of silver oxide. In the present study, we investigated the molecular mechanism of anti-metastasis activity of TEG derivatives using in silico and cell-based in vitro analyses.

## 2. Results

### 2.1. TEG Derivatives Inhibited the Migration and Invasion Potential of Cancer Cells

**Figure 1:**
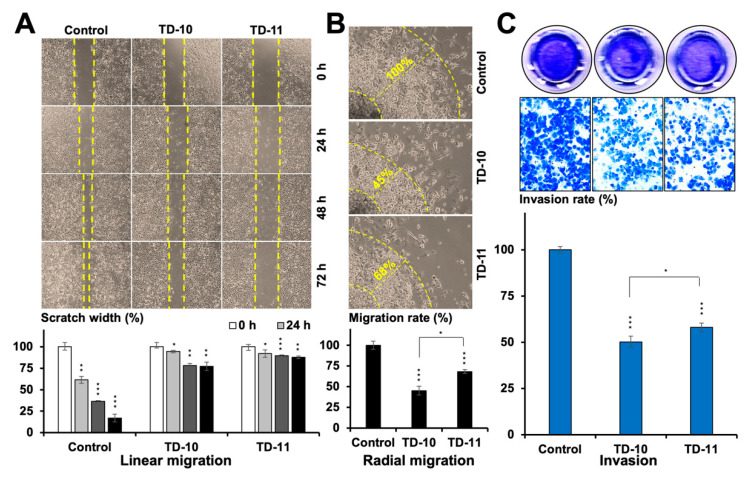
Anti-metastatic activity of the TEG derivatives in Panc-1 cells. (**A**) Wound scratch migration assay showed a delay in primary tumor site migration potential over a period of 72 h. (**B**) Radial migration assay showed an inhibition of the distension migration from the secondary tumor site over a period of 72 h. (**C**) Matrigel^®^ invasion assay showed an anti-invasive potential of the derivatives over a period of 24 h. Statistical significance was defined as *p*-values (*) where * < 0.05, ** < 0.01 and *** < 0.001 represent significant, very significant and highly significant, respectively.

In order to establish the effect of TD-10 and TD-11 on cell migration, we first selected their non-toxic doses by MTT-based dose titration assay in Panc-1, MDA-MB-231, HeLa, DLD-1, T.T and HSC3 cells (Figure S1). A low (nontoxic) dose (IC_10_ = 0.005% for both TD-10 and TD-11) was used in cell migration (linear and radial migration) and invasion assays. As shown in Figure 1A,B and Figure S2, the non-toxic doses of TD-10 and TD-11 caused a significant delay in the migration of Panc-1, MDA-MB-231 and HeLa cells. Remarkable reduction (~50%) in invasion was recorded (Figure 1C). Of note, the anti-migratory (radial) and anti-invasive effect of TD-10 was comparatively stronger than that of TD-11. Based on these results, we examined the interactions of TD-10 and TD-11 with proteins involved in the regulation of migration and invasion capacity of cells.

### 2.2. Molecular Docking and Experimental Analysis of the Effect of TD-10 and TD-11 on VEGFA-VEGFR Complexes

**Figure 2:**
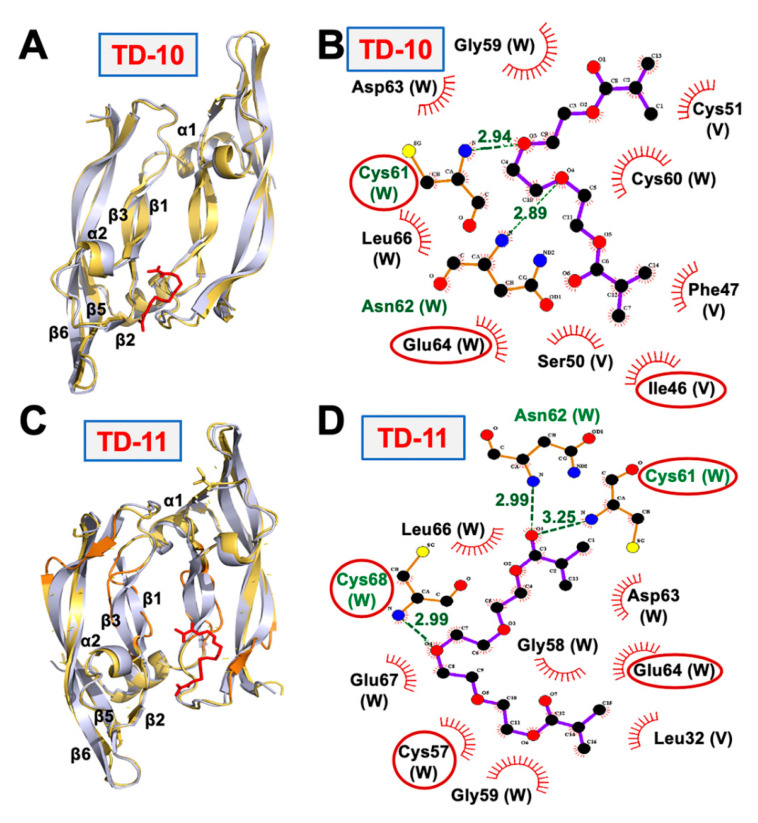
Superimposition of the structure of VEGFA protein dimer (grey) with VEGFA-TD-10 (yellow-red) complex (**A**) and VEGFA-TD-11 (yellow-red) Complex (**C**) interactions formed by TD-10 (**B**) and TD-11 (**D**) with VEGFA protein receptor-interacting interface. Interactions formed with key interacting residues are highlighted in red circles.

**Figure 3:**
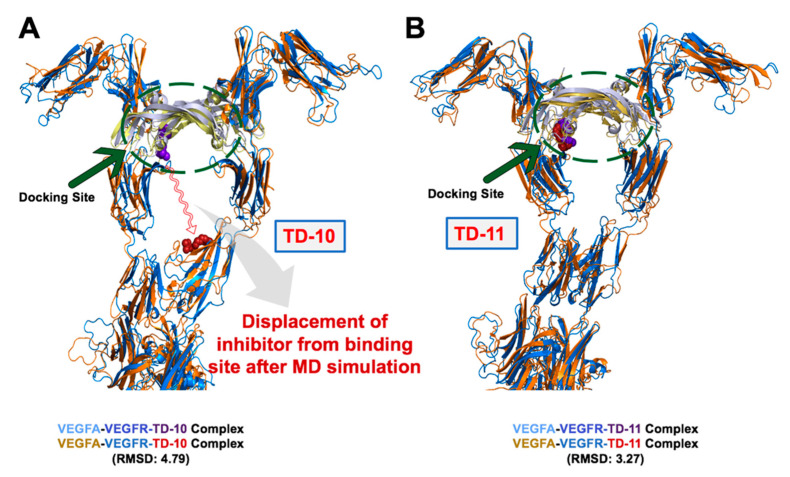
Superimposed structure of (**A**) VEGFA-VEGFR-1-TD-10 docked complex (grey-blue-purple) with VEGFA-VEGFR-1-TD-10 simulated structure (yellow-orange-red) Complex. (**B)** VEGFA-VEGFR-1-TD-11 docked complex (grey-blue-purple) with VEGFA-VEGFR-1-TD-11 simulated complex (yellow-orange-red).

TD-10 and TD-11 were docked to the key interacting residues of both VEGFA homodimer and VEGFA-VEGFR-1 heterodimer complexes [40,41]. Both TD-10 and TD-11 formed hydrogen bonds and hydrophobic interactions with key interacting residues of the VEGFA receptor (highlighted in red circles in Figure 2B,D), suggesting that they could interfere with the interaction of VEGFA and its receptor. TD-11, but not TD-10, also caused local disruption of the secondary structure of VEGFA protein dimer (Figure 2A,C). Docking of TD-10 and TD-11 to VEGFA-VEGFR heterodimer complex revealed that TD-11 was stable at the interaction interface, and no change in the secondary structure of VEGFA was induced in VEGFA-VEGFR complex (Figure 3). The binding energy of VEGFA protein with its receptor, VEGFR, was also slightly reduced from −24.2 kCal/mol to −20.9 kCal/mol and −22.4 kCal/mol in case of TD-11 and TD-10, respectively. These data predicted that TD-11, but not TD-10, is capable of disrupting the secondary structure of VEGFA protein and thereby may interfere with its interaction with VEGFR. On the other hand, TD-11 was not able to either induce a change in VEGFA structure in VEGFA-VEGFR complex or disrupt already assembled complex.

**Figure 4:**
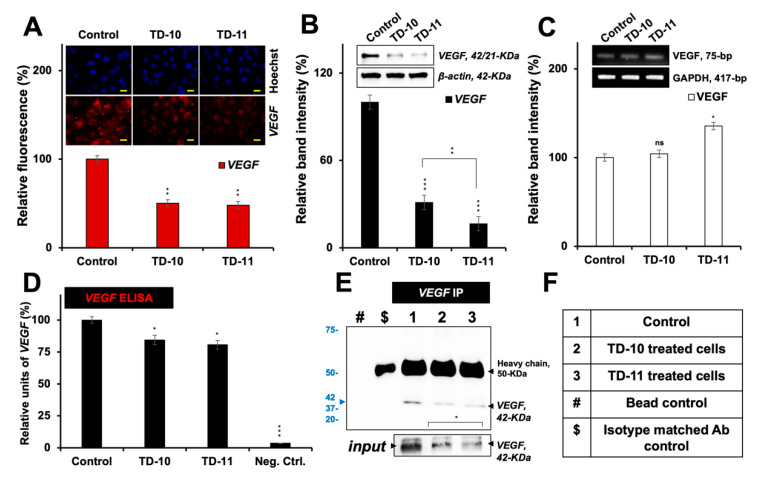
VEGF analyses in Panc-1 cells treated with TD-10 and TD-11. Immunostaining (**A**), Western blotting (**B**) and RT-PCR (**C**) showed downregulation of VEGF expression at the protein (**A**,**B**) and mRNA (**C**) levels, respectively. ELISA (**D**) and immunoprecipitation (**E**,**F**) assays showed downregulation of secreted VEGF expression in the surrounding culture medium. Scale 20 µm. Statistical significance was defined as *p*-values (*) where ns, * < 0.05, ** < 0.01 and *** < 0.001 represent not significant, significant, very significant and highly significant, respectively.

We next examined the expression of VEGF by immunostaining, Western blotting and RT-PCR in control and treated cells. VEGF was found to be significantly downregulated at the protein level (as detected by immunostaining and Western blotting with specific anti-VEGF antibodies) in Panc-1, MDA-MB-231 and HeLa cells treated with nontoxic doses of both TD-10 and TD-11 (Figure 4A,B, Figures S3 and S4). Of note, no decrease in the VEGF transcript was detected either in TD-10- or TD-11-treated cells (Figure 4C and Figure S5). Change in the protein level was also endorsed by ELISA (Figure 4D) and immunoprecipitation (Figure 4E,F) assays performed on the cultured growth medium to detect secreted VEGF. Downregulation of protein in all the assays was stronger in TD-11- than in TD-10-treated cells; the results were in line with the data from in silico analysis that showed local disruption of the protein dimer structure with TD-11. Taken together with the RT-PCR data, it may be concluded that TD-10 and TD-11 inhibit VEGF by disrupting the structure of protein dimers, and TD-11 causes stronger inhibition. However, the observation did not completely align with the migration assays (Figure 1) that revealed a stronger effect of TD-10. Since migration of cells is tightly controlled by the expression of MMPs, we next investigated the effect of TD-10 and TD-11 on MMPs by molecular docking and experimental assays.

### 2.3. Molecular Docking and Experimental Analyses of Interactions of TD-10 and TD-11 with MMP-2, MMP-7 and MMP-9

The catalytic domain of the MMP family of proteins was targeted to examine the potential of TEG derivatives as inhibitors. The catalytic domain of MMPs comprised five β sheets (βI-βV), three alpha helices (αA-αC), long and flexible Ω-loop connecting αB and αC helices and S-loop connecting βIII and βIV sheets. The βIV strand, a small fragment of S-loop and Ω-loop forms the catalytic cleft along with a catalytic Zn ion, coordinated by His403, His407 and His413 in an active state. The catalytic cleft is comprised of six binding subsites S3-S3’ along its length [42]; among them, S1’ pocket offers more variability in terms of length and properties, is less solvent-exposed and has been a focus of interest to achieve selective inhibition [43]. The S1’ pocket of MMP-2 and MMP-9 is classified as a large pocket due to the presence of Leu at position 399, whereas in the case of MMP-7, Leu is replaced by the presence of large amino acid residue, Tyr. Presence of Tyr at position 399 leads to smaller pocket size in MMP-7. Both TD-10 and TD-11 were docked at S1’ pocket (residues 423–433) of MMP-2, MMP-7 and MMP-9.

**Figure 5:**
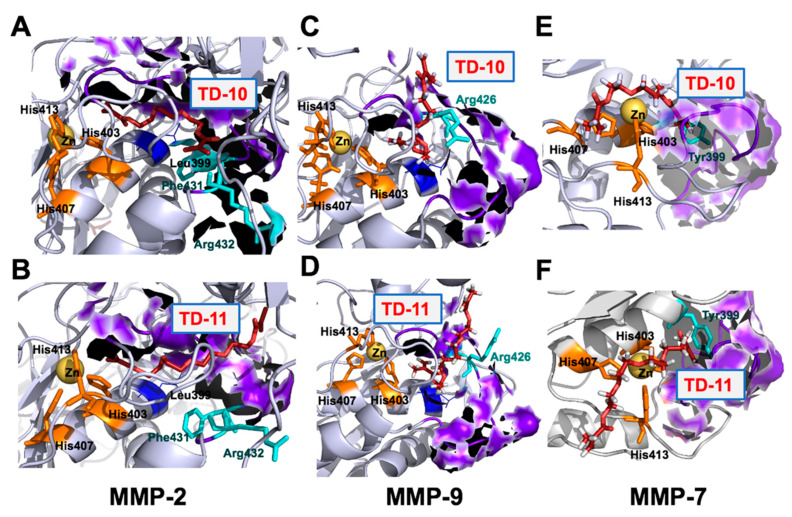
Interaction of TD-10 and TD-11 with S1’ pocket of MMP-2 (**A**,**B**), MMP-9 (**C**,**D**) and MMP-7 (**E**,**F**), respectively. S1’ pocket residues surfaces are highlighted in purple color and gatekeeper residues of S1’ pocket shown in cyan color. Three histidine residues that coordinate catalytic Zn ion are shown in orange color.

**Figure 6:**
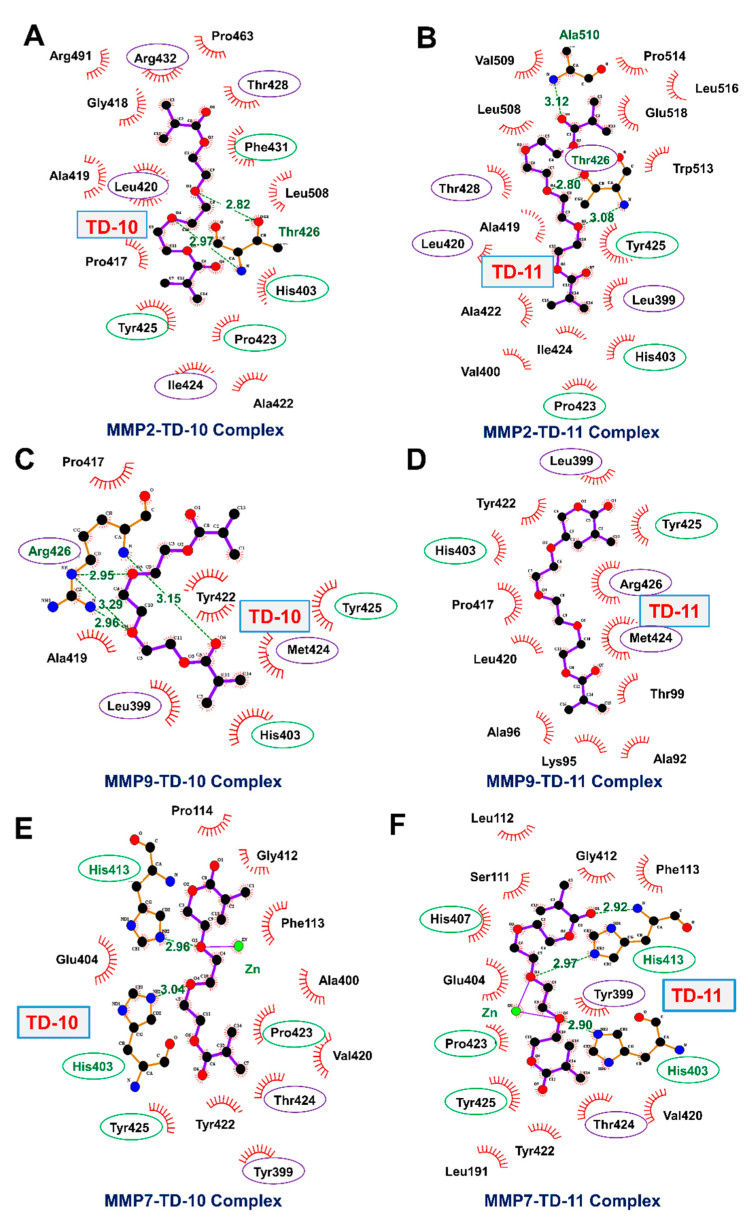
Diagram showing interactions formed by TD-10 and TD-11 with S1’ pocket of MMP-2 (**A**,**B**), MMP-9 (**C**,**D**) and MMP-7 (**E**,**F**), respectively. S1’ pocket residues are highlighted with purple color circle, and conserved residues are highlighted with green circles (residues naming has been done corresponding to MMP-2 protein structure (1CK7)).

MMP-2 and MMP-9 belong to the same metalloproteinase family (gelatinases), and their mode of interactions with TD-10 and TD-11 were found to be similar. Both TD-10 and TD-11 were not chelating with catalytic Zn ion coordinated by three His residues (His403, His407 and His413) near S1’ pocket and were showing interactions with the specificity loop of MMP-2 and MMP-9 (Figure 5A,D and Figure 6A,D). The detailed analyses of interactions formed by TD-10 and TD-11 at the S1’ pocket were performed to see if these molecules were able to induce the open state of the S1’ pocket as reported in literature [43]. In the case of MMP-2, a shift in the position of amino acid residues, Phe431 and Arg432, is reported in the presence of inhibitor to open up the S1’ pocket and facilitate their entry [43]. In the case of TD-10 and TD-11, a shift in the position of these two important residues of MMP-2 was not observed, and thereby open state of S1’ pocket was not induced (Figure 5A,B). However, both molecules showed significant hydrogen bond and hydrophobic interactions with S1’ pocket residues of MMP-2 (Figure 6A,B). TD-10 showed hydrophobic interactions with Phe341 and Arg432 in an attempt to enter into the S1’ pocket but hindered by the orientation of Phe341 and Arg432 (Figure 5A and Figure 6A). TD-11, on the other hand, instead of trying to enter into the interior of S1’ pocket of MMP-2 by shifting Phe341 and Arg432, makes its way through S1’ pocket by interacting with outer surface residues of the pocket (Figure 5B). TD-11 binds more strongly at the interaction site of MMP-2 by forming more hydrogen bond and hydrophobic interactions, with a binding energy of -74.81 kCal/mol as compared to -63.55 kCal/mol binding energy of TD-10 (Table 1).

In case of MMP-9, Arg 426 acts as a gatekeeper residue and regulates the opening of S1’ pocket by moving outwards and orients towards the cavity to close it [43]. TD-10 was not able to induce outward movement of Arg426, and therefore could not make its entry into the hydrophobic cavity of S1’ pocket (Figure 5C). Instead, it formed a hydrogen bond with Arg426 and hydrophobic interactions with wall-forming-segment residues (Met424 and Tyr425) of S1’ pocket and found its way outside via aligning longitudinally from the middle of coordinated catalytic Zn ion and S1’ pocket (Figure 5C and Table 1) [43]. Similarly, in the case of TD-11, the molecule was not able to enter the interior of S1’ pocket and align itself longitudinally between the wall-forming segment of S1’ pocket and catalytic Zn ion (Figure 5D). However, TD-11 had induced the open state of S1’ pocket by flipping Arg426 in outward direction but still preferred the same orientation of interaction as formed by TD-10 in the closed state of S1’ pocket (Figure 5D). TD-10 molecule bound more strongly with MMP-9 at its interaction site with a binding energy of −58.44 kCal/mol, whereas TD-11, in spite of forming more hydrophobic interactions in comparison to TD-10, showed binding energy of −45.78 kCal/mol only (Figure 6C,D and Table 1).

MMP-7 belongs to the family of matrilysins, characterized by a small S1’ pocket due to presence of large amino acid residue at position 399 (Tyr). Tyr399 is mobile and can rotate to induce an open state of S1’ pocket [43]. Both TD-10 and TD-11 could not induce open conformation of S1’ pocket and instead interacted hydrophobically with Tyr399 residue and wall-forming-segment residues (Pro423, Thr424 and Tyr425) of S1’ pocket of MMP-7 protein (Figure 6E,F). It was observed that TD-10 and TD-11 serve as catalytic inhibitors by interacting with catalytic Zn ion along with the formation of hydrogen bonds and hydrophobic interactions with three conserved His residues that coordinate catalytic Zn ion (Figure 5E,F and Figure 6E,F). TD-11 showed strong binding with the energy of −28.97 kCal/mol as compared to TD-10, which had a binding energy of −15.86 kCal/mol. Based on these analyses, it was predicted that both TD-10 and TD-11 may exert their anti-metastatic activity by targeting S1’ pocket of MMP-2 and MMP-9. Although they do not completely fit into the S1’ pocket of these proteins, they interact stably with wall-forming-segment residues of the S1’ pocket. In the case of MMP-7, they acted by serving as Zn ion chelators by interacting with Zn ions and His residues coordinating Zn ion.

**Figure 7:**
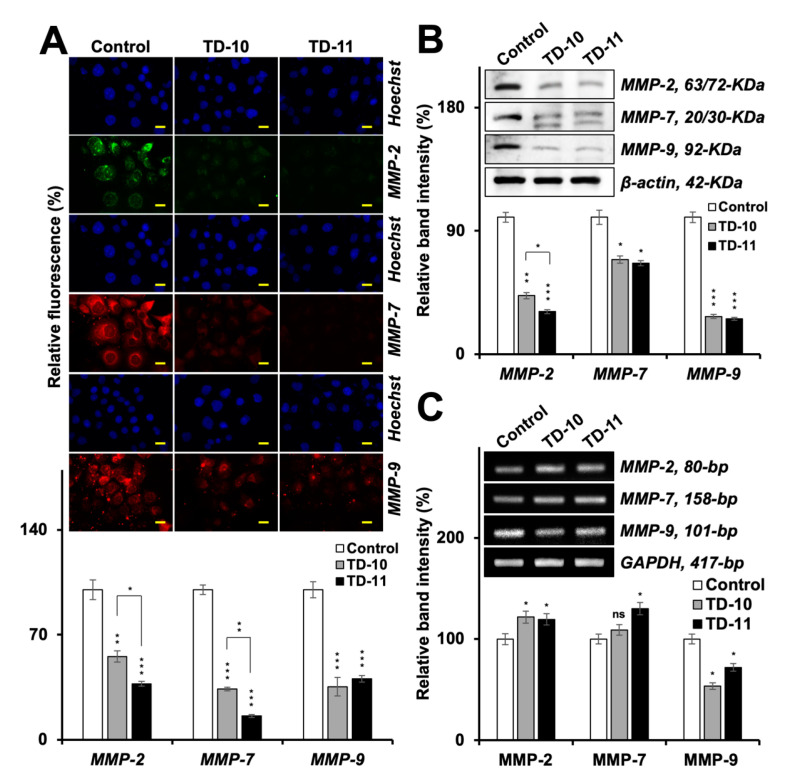
Analyses of MMPs in Panc-1 cells treated with TD-10 and TD-11. Immunostaining (**A**), Western blotting (**B**) and RT-PCR (**C**) showed downregulation of MMP-2 and MMP-7 proteins and inhibition of both protein and transcription of MMP-9. Scale 20 µm. Statistical significance was defined as *p*-values (*) where ns, * < 0.05, ** < 0.01 and *** < 0.001 represent not significant, significant, very significant and highly significant, respectively.

We next examined the expression of MMP-2, -7 and -9 proteins in control and treated cells by immunostaining and Western blotting using specific antibodies (Figure 7A,B, Figures S3 and S4). Immunostaining revealed downregulation in TD-10- and TD-11-treated cells; the latter was relatively stronger for MMP-2 and -7 and caused a stronger decrease in MMP-7 as compared to MMP-2. Western blotting revealed stronger inhibition of MMP-2 and -9 in TD-10/-11-treated cells and a weak effect on MMP-7. Of note, amongst the three MMPs, MMP-9 expression showed the strongest decrease by Western blotting and also tracked to transcript level (Figure 7B,C). Since MMPs and VEGF are highly expressed in cancer cells and contribute to their metastatic potential, their decrease in TD-10- and TD-11-treated cells are deemed valuable for cancer therapeutics. In order to further dissect their mechanisms of action, we investigated EMT regulatory proteins that are also linked to VEGF and MMPs.

### 2.4. Molecular Docking and Experimental Analyses of Interactions of TD-10 and TD-11 with Vimentin and Mortalin

Amongst several proteins that regulate EMT (epithelial-to-mesenchymal transformation), we examined interactions of TD-10 and TD-11 with hnRNP-K, vimentin, p53 and mortalin. The ssDNA/RNA binding domain of hnRNP-K molecule was targeted to check if they can inhibit RNA binding ability of the protein [44]. It was observed that both TD-10 and TD-11 did not show binding with RNA/ssDNA-interacting residues (Table S1). The binding of TEG derivatives around Cys328 residue of vimentin tetramer was also explored to check if TD-10 and TD-11 have the capability to disrupt vimentin filaments assembly. Cys328 is a critical residue that interacts with Zn ion and plays a key role in vimentin assembly and its reorganization in response to various oxidants [45]. TD-10 was unable to show binding around Cys328, while TD-11 showed very weak binding at the interaction site (Table S1). Vimentin-TD-11 complex was simulated to check the stability of TD-11 at the binding site. It was observed that TD-11 could not stably interact with vimentin tetramer due to very weak binding energy and eventually lost its interaction with the protein. Next, the effect of TD-10 and TD-11 on p53-mortalin interaction was examined by targeting the interacting residues of both the proteins. Mortalin-interacting residues, amino acid residues 323 to 327, of p53 protein were targeted. Among the two inhibitors, only TD-11 was able to show binding at the mortalin-interacting site of p53 protein (Table S1). Similarly, p53 interacting amino acid residues (253 to 282) of mortalin protein were targeted to check the binding of TEG derivatives. Yet again, only TD-11 showed interaction with p53-interacting domain of mortalin (Table S1). p53-TD-11 complex was simulated, and docking of mortalin with p53 protein in the presence and absence of TD-11 was carried out to explore the effect of TD-11 on p53-mortalin complex formation. The protein–protein docking was performed around p53-interacting residues of mortalin and mortalin-interacting residues of p53 using HADDOCK webserver. The binding energy of p53 and mortalin in p53-mortalin complex was found to be −87.5 ± 4.0 kCal/mol, whereas it was reduced to −76.1 ± 3.7 kCal/mol in p53-TD-11-mortalin complex. Protein-protein binding energy of p53-mortalin and p53-TD-11-mortalin complexes were also calculated using Prodigy webserver; once again, a very slight reduction in the binding energy of p53 and mortalin was observed in the presence of TD-11: from −8.6 to −7.8 kCal/mol. Hence, it may be concluded from this analysis that both TD-10 and TD-11 could not inhibit p53-mortalin interaction, hnRNP-K-RNA/ssDNA interaction and vimentin filament assembly.

**Figure 8:**
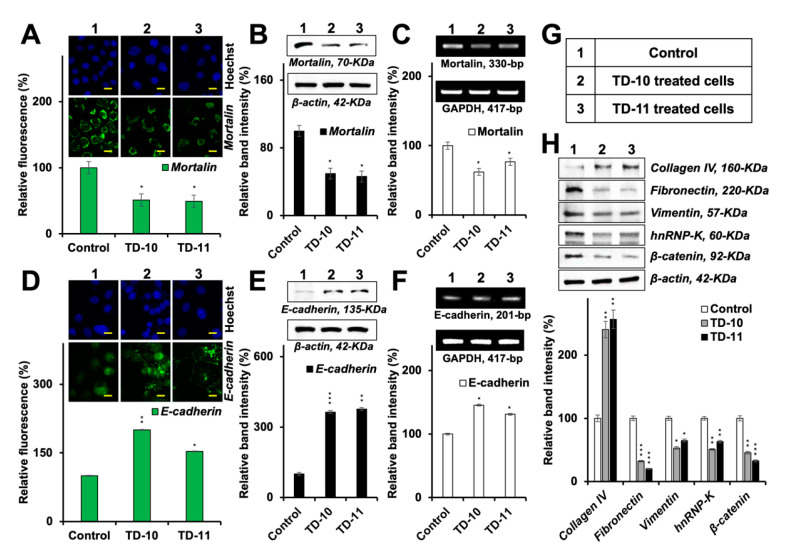
Downregulation of mortalin in response to the treatment with TD-10 and TD-11 in Panc-1 cells. Immunostaining (**A**) and Western blotting (**B**) showed decrease in mortalin protein. (**C**) RT-PCR showed downregulation of expression of mortalin at the transcription level. Immunostaining (**D**) and Western blotting (**E**) showed an increase in E-cadherin protein. (**F**) RT-PCR showed upregulation of E-cadherin at the transcription level. (**G**) List of treatments in A–F is shown. (**H**) Western blotting and quantitation showed upregulation of collagen IV and downregulation of other EMT proteins in cells treated with the TEG derivatives. Scale 20 µm. Statistical significance was defined as *p*-values (*) where * < 0.05, ** < 0.01 and *** < 0.001 represent significant, very significant and highly significant, respectively.

**Figure 9:**
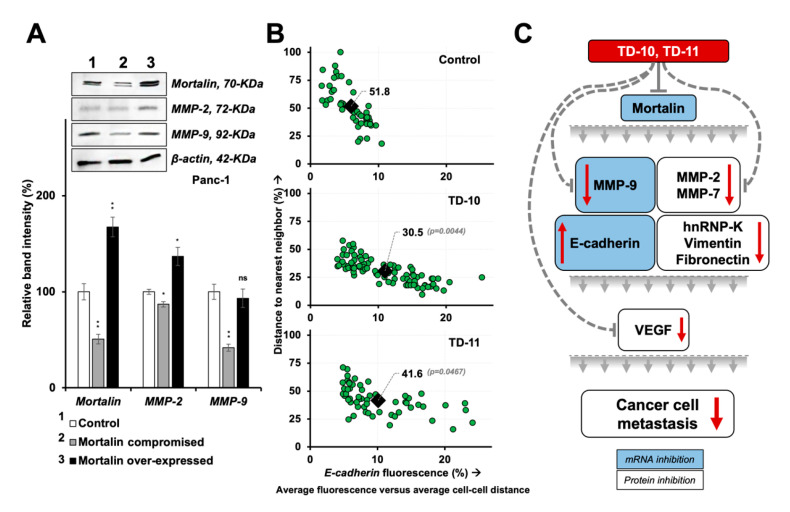
Dependency of EMT on mortalin expression and re-epithelization of the highly metastatic Panc-1 cells treated with TEG derivatives. (**A**) Western blotting and quantitation in mortalin-compromised and -over-expressed cells showed reduction and increase in MMP expression, respectively. (**B**) Cell congregation analyses showed that E-cadherin fluorescence had an inverse relation with the distance between the expressing cells and the TEG derivatives that caused the epithelial transformation of these cells. (**C**) Line diagram summarizing the mechanism of mortalin-dependent anti-metastasis activity of the TEG derivatives, TD-10 and TD-11. Transcriptional changes are highlighted in blue. Statistical significance was defined as *p*-values (*) where * < 0.05 and ** < 0.01 represent significant, very significant and highly significant, respectively.

Mortalin has earlier been shown to act as one of the key regulators of EMT [46]. Whereas its over-expression enhances the migration and invasion capability of cells and increases the expression of vimentin, fibronectin, β-catenin, α-SMA, CK-14 and hnRNP-K, its knockdown had been associated with downregulation of these proteins and reversal of EMT [46]. Lung cancer cells treated with TD-10 and TD-11 also showed a decrease in mortalin expression [39]. In light of this information, we examined the expression of mortalin in TD-10 and TD-11 treated Panc-1 cells. As shown in Figure 8A,B, mortalin protein was significantly downregulated in treated cells. Of note, in addition to the downregulation at the protein level as endorsed by immunostaining and Western blotting, we detected a significant decrease in mortalin mRNA in treated cells (Figure 8C). These data supported the decrease in mortalin protein in premises of in silico results that showed poor interactions with TD-10 and TD-11 (binding energy of −0.307 and −2.348 kCal/mol, respectively) with mortalin (Table S1). Furthermore, transcriptional repression of mortalin was consistent and significantly stronger by TD-10 than TD-11 (Figure 8C), and that matched with the effect seen in migration and invasion assays (Figure 1). We also examined the expression of downstream effectors of mortalin involved in EMT. As shown in Figure 8D–G, consistent and proportional upregulation of E-cadherin protein and mRNA was observed in TD-10/-11 treated cells. Proteins that promote mesenchymal transformation (fibronectin, vimentin, hnRNP-K, β-catenin) showed a decrease (Figure 8H) in TD-10- and TD-11-treated cells. On the other hand, collagen IV protein, one of the degradation targets of several MMPs [21], showed an increase. Since these changes seemed consistent with the changes in mortalin in TD-10 and TD-11 treated cells, we next generated mortalin-over-expressing and -compromised cells. Analyses of MMP-2 and MMP-9 proteins revealed that in mortalin-compromised cells, MMP-9 was affected the most and showed a remarkable decrease (Figure 9A). On the other hand, over-expression of mortalin caused an increase only in MMP-2. These data supported the effects of a decrease in mortalin at mRNA and protein levels in TD-10- and TD-11-treated cells. We next examined if such a decrease in mortalin and MMP-9 was sufficient to reverse the EMT signaling and determined intercellular distance that is translated to the cell–cell interactions and cell adhesions (factors active in the epithelial cell phenotype) [47,48]. As shown in Figure 9B, E-cadherin expression was laid inversely to the distance between the cells, indicating that the TEG derivatives induced epithelial phenotype in these cells. The effect was significant for both the derivatives, yet stronger for TD-10 than for TD-11. Thus, taken together, our results suggested that the TEG derivatives TD-10 and TD-11 have multimode actions: in addition to the targeting of VEGF and MMPs, they possess the potential to transcriptionally suppress mortalin and MMP-9, leading to the reversal of EMT and epithelialization of the metastatic cancer cells.

## 3. Discussion

Pancreatic cancer (Panc-1) cells have previously been shown to (i) abundantly express mortalin, VEGF and MMP [49,50,51]; (ii) harbor a high number of oncogenic mutations in KRAS, TP53, CDKN2A/p16 and SMAD4/DPC4 [52]; (iii) possess strong migratory and adhesive abilities [53] and (iv) easily cluster and difficultly differentiate into their functional phenotypes [54]. Thus, we chose to examine the regulation of EMT by the TEG derivatives in these cells. Migratory and invasive cancers are characterized by their mesenchymal phenotype. The mesenchymal cells are extracellularly attached and embedded in the tissues through a complex scaffold of proteins made up of collagen, fibronectin and integrins. MMPs collectively with other proteins aid in the migratory nature of cancer cells bundled through a process called EMT. Widely studied and explored, one of the key proteins confining cells from EMT is E-cadherin. Cadherins are type-1 transmembrane cell adhesion molecules that form an essential part of adherens junctions (AJs). Its extracellular domain interacts with the extracellular components of E-cadherin of neighboring cells, forming a tight labyrinth-like lattice, while the intracellular domain is widely known to interact and bind to β-catenin. Mortalin has been shown to promote cell proliferation and invasion via induction of EMT and regulate the expression of E-cadherin [46,55]. The relevance of mortalin to other EMT proteins such as MMPs and angiogenesis-specific permeability factors such as VEGF is largely unknown. In the present study, we demonstrate that the TEG derivatives transcriptionally downregulate the expression of mortalin (Figure 8), followed by a dramatic reversal of EMT through significant changes in the hallmark proteins.

MMPs (especially -2 and -9) are known to degrade collagen IV [21], enabling the release of cells from their original (primary) niche and allowing them to migrate to distant tissues through local and systemic circulations. Specific roles of MMPs have been well discovered and documented. In mesenchymal cells, MMP-2 was shown to cleave fibronectin into smaller fragments and thereby increase the chances of these circulating cancer cells to attach to the secondary sites [56]. MMP-9 lies downstream of TGF-β1 and is involved in Slug/EMT induction through the shedding of E-cadherin [57]. As an interplay, it disrupts AJs to reduce the E-cadherin level and degrade into its soluble form [58,59]. MMP-9 expression, reciprocally, has been shown to be induced by fibronectin via involvement of integrin receptor α5β1 [60,61,62]. We found that the TEG derivatives targeted MMP-2 and MMP-9 and upregulated collagen IV expression and downregulated fibronectin expression in Panc-1 cells (Figure 7 and Figure 8H). The mechanism of action of TD-10 and TD-11 on MMP-2, MMP-9 and MMP-7 was studied using in silico approaches by targeting the catalytic domain of these proteins. It was observed that TD-10 and TD-11 have the capability to serve as catalytic domain inhibitors of MMP-2, MMP-7 and MMP-9. Both TD-10 and TD-11 targeted S1’ Pocket of MMP-2 and MMP-9, but also the catalytic Zn ion binding site of MMP-7. Among the two, TD-11 showed stronger binding to MMP-2 and MMP-7 through hydrogen bonds and hydrophobic interactions. The functionally diverse protein vimentin has also been reported to be broken down by MMPs and other proteases from a large filamentous form into smaller units within the process of EMT. This causes a homogenous distribution of the vimentin protein and triggers its over-expression, thereby controlling the organization and structure and function of the cell–matrix adhesion [63]. The expression of vimentin promotes mesenchymal morphology of the metastatic cancer cells. It also regulates acto-myosin contractile forces responsible for cell motility. Although the TEG derivatives decreased the expression of vimentin in cancer cells (Figure 8H), they could not directly interact with the protein. Metastasis-specific proteins hnRNP-K and β-catenin were found to be decreased in response to the treatment with TD-10 and TD-11, consistent with our previous findings [39]. Cancer cell invasion has been shown to be promoted by TNFα via MMP-9 expression, thus activating EMT [64]. TNFα, on several occasions, has been shown to regulate MMP expression to contribute to local inflammation via activation of the NF-κB pathway, essential for wound healing [21]. MMPs are tightly regulated. MMP-7 has been suggested to increase the IGF bioavailability and insulin function via its proteinase activity on IGFBP-3. Increased insulin function has been shown to activate PI3K/Akt signaling, thereby inhibiting the expression of MMP-9 protein [65,66]. MMP-2 and -7 seemed not to be downregulated by the TEG derivatives at the transcriptional level; however, MMP-9 did show significant inhibition in the cell mRNA by the TEG derivatives (Figure 7C). Whereas the extracellular domain of E-cadherin has been shown to be a target of MMP-7-mediated insults [67], Liu et al. (2018) found that the Wnt/β-catenin signaling pathway inversely regulates the expression of MMP-2 and MMP-9 proteins [68].

Belotti et al. (2003) showed that MMP-2 and MMP-9 possess and maintain the ability to induce the release of biological active VEGF [69]. VEGFA from the VEGF family is known to direct the proliferation and migration of the endothelial cells and their alignment to form vasculature and fenestrations. The molecular docking and molecular dynamics analyses of the effect of TEG-derivatives on VEGFA dimer revealed that TD-11 caused local disruption of the secondary structure of protein, while no such changes were observed in the case of TD-10 binding (Figure 2A,C). Similarly, only TD-11 showed stable binding at the receptor-interacting domain of VEGFA in VEGFA-VEGFR complex; however, it completely failed to disrupt the complex, and no structural change in VEGFA was induced by TD-11 when in complex with its receptor (Figure 3). MMP-9 is reported to be commonly over-expressed in metastatic cancer cells, progenitor cells, local inflammatory infections, acute hypoxia and physical trauma; its expression is regulated by VEGF through the expression of ETS-1 bound to the MMP-9 gene promoter [70]. Thus, the transcriptional downregulation of MMP-9 downstream to VEGF was justified (Figure 7C). Similar to Panc-1 cells, breast (MDA-MB-231), cervical (HeLa), colorectal (DLD-1), esophageal (T.T) and tongue (HSC3) cancer cell lines treated with non-toxic doses of TD-10 and TD-11 (Figure S1) showed a significant delay in cell migration (Figure S2), and a decrease in VEGF and MMPs protein expression (Figures S3 and S4). Only MMP-9 showed a decrease at the transcriptional level (Figure S5). MMPs and VEGF must not be confused as the oncoproteins, as the former are crucial for cell migration and neoangiogenesis as a part of embryogenesis, organ development and ordinary tissue repair. Their complete abolishment may be catastrophic as it has been with a number of drugs in the clinic. Thus, the molecules that may moderately suppress their activity should be favored and tried more frequently. We show that the TEG derivatives TD-10 and TD-11 at their non-toxic doses, via mortalin inhibition, reasonably inhibit the expression of MMPs, VEGF and a host of other metastasis-escalating proteins. Their use as anti-mortalin anticancer therapeutic molecules is recommended and the human-based trials are warranted.

## 4. Materials and Methods

### 4.1. Cell Line and Reagents

Panc-1 (pancreatic adenocarcinoma), MDA-MB-231 (breast adenocarcinoma), HeLa (cervical adenocarcinoma), DLD-1 (colorectal adenocarcinoma), T.T. (esophageal squamous cell carcinoma) and HSC3 (oral squamous cell carcinoma) cells were obtained from the Japanese Collection of Research Bioresources (JCRB), Japan and cultured in Dulbecco’s modified Eagle’s medium (Invitrogen) supplemented with 5% fetal bovine serum and 1% penicillin/streptomycin in a humidified incubator (37 °C and 5% CO_2_). TEG derivatives TD-10 or triethylene glycol dimethacrylate (261548-250ML) and TD-11 or tetraethylene glycol dimethacrylate (86680-100ML) were purchased from Sigma-Aldrich (St. Louis, MO, USA). Primary antibodies against VEGF (Santa Cruz, Dallas, TX, USA, SC-507), β-actin (AbCam, Cambridge, UK, ab49900), MMP-2 (Santa Cruz, Dallas, TX, USA, SC-13594), MMP-7 (Santa Cruz, Dallas, TX, USA, SC-30071), MMP-9 (AbCam, Cambridge, UK, ab38898), Mortalin [71], E-cadherin (Cell Signaling, Denvers, MA, USA, 5296S), Collagen IV (AbCam, Cambridge, UK, ab6586), Fibronectin (Santa Cruz, Dallas, TX, USA, SC-52331), Vimentin (Santa Cruz, Dallas, TX, USA, SC-6260), hnRNP-K (Cell Signaling, Denvers, MA, USA, 4675S) and β-catenin (Santa Cruz, Dallas, TX, USA, SC-7963) were used in immunostaining and Western blotting.

### 4.2. Dose Titration

Two thousand cells per well were seeded in 96-well plates, allowed to settle overnight, and then treated with varying doses of the TEG derivatives. The control or treated cells were incubated for 24 h followed by the addition of 10 µL of phosphate-buffered saline (PBS) containing 5 mg/mL MTT (3-(4,5-dimethylthiazol-2-yl)-2,5-diphenyltetrazolium bromide) (Life Technologies, Carlsbad, CA, USA, M6494), followed by further incubation for 4 h. The culture medium containing MTT was replaced with DMSO (Wako, Osaka, Japan, 043-07211) and mixed thoroughly. The optical density was measured at 570 nm using Tecan infinite M200^®^ Pro microplate reader (Tecan Group Ltd., Mannedorf, Switzerland). Cell viability was calculated as a percentage against the control, and IC values (IC_10_ and IC_50_) were determined using Microsoft™ Office© 2016.

### 4.3. Cell Migration and Invasion Assays

Wound scratch migration assay: 25 × 10^4^ cells per well were seeded in 6-well plates and allowed to settle overnight. Cells were then uniformly scratched with the help of a pipette tip, washed thoroughly and treated with varying doses of the TEG derivatives for 72 h. Cell photographs were taken every 24 h by a phase-contrast microscope. The girth of the gap (scratch) was calculated using ImageJ software (NIH, Bathesda, MD, USA, 1.52a) and tabulated as a percentage against the control using Microsoft™ Office© 2016.

Radial migration assay: Panc-1 tumors were made by hanging drop method as described earlier [72]. Adhered tumors were treated with varying doses of the TEG derivatives for 72 h. Cell photographs were taken on the fifth day under a phase-contrast microscope. The length to which the cells in the tumor radially migrated was calculated using ImageJ software (NIH, Bathesda, MD, USA, 1.52a) and tabulated as a percentage against the control using Microsoft™ Office© 2016.

Matrigel^®^ invasion assay: Invasion assay was carried out using BioCoat Matrigel^®^ Invasion kit (Corning, NY, USA, 3-354480), and 5 × 10^4^ cells suspended in 0.5 mL serum-free Dulbecco’s modified Eagle’s medium (DMEM, Wako, Osaka, Japan, 041-29775), were plated into the top of invasion inserts with/without the TEG derivatives. The bottom well of a 24-well plate was filled with 0.75 mL DMEM supplemented with 10% fetal bovine serum. After 24 h, the inserts were transferred to fresh plates and washed thrice with PBS. Cells suspended in the Matrigel^®^ basement membrane matrix at the bottom of each insert were fixed in methanol: acetone (1:1) and stained with 0.5% Crystal Violet overnight. The excess stain was removed by washing with ultrapure water to remove the excess stain. The inserts were air-dried, visualized under the microscope, photographed and de-stained to determine absorbance. Histograms representing the results were plotted using Microsoft™ Office© 2016.

### 4.4. Molecular Docking and Simulations to Check the Effect of TD-10 and TD-11 with MMPs (MMP-2, MMP-7 and MMP-9) Family of Protein and VEGFA Protein and VEGFA-VEGFR-1 Complex

Crystal structure of MMP family of proteins, MMP-2 (PDB Id: 1CK7), MMP-7 (PDB ID: 2Y6C) and MMP-9 (PDB ID: 5UE3) were obtained from Protein Data Bank [73,74,75]. The X-ray crystallography structure of VEGFA protein in complex with extracellular domain (ECD) of its receptor was also obtained from Protein Data Bank (PDB ID: 5T89) [76]. The coordinates of the structure of VEGFA was obtained from VEGFA–VEGFR-1 complex. The structures of VEGFA, VEGFA-VEGFR-1 complex, MMP-2, MMP-7 and MMP-9 were prepared using PrepWizard module of Schrodinger 2018-4. The structures of ligands, TD-10 and TD-11, were drawn using 2D sketcher panel and prepared using LigPrep module of Schrodinger suite 2018-4 version. Glide extra precision (XP) algorithm was used to perform docking of ligands at the S1’ Pocket of MMP class of proteins [77,78]. In the case of VEGFA protein, its receptor-interacting residues were targeted by generating a grid around nine residues of VEGFA, Phe17, Ile43, Ile46, Glu64, Gln79, Ile83, Lys84 and Pro85, which play a key role in interaction with receptor [40,41]. Docking of ligands was performed around these nine receptor-interacting residues with both VEGFA protein and VEGFA-VEGFR-1 complex.

The effect of TD-10 and TD-11 was also studied on other EMT regulatory proteins, including hnRNP-K (PDB ID: 1ZZI) [79], vimentin tetramer (computationally modelled structure) [80], p53 (PDB ID: 1OLG) [81] and mortalin (PDB ID: 4KBO) [82] using molecular docking and molecular dynamic simulations. Protein–protein docking was performed to generate p53-mortlain complex using HADDOCK webserver, and protein–protein binding energy was calculated using PRODIGY webserver [83,84].

The docked complexes were simulated to monitor the stability of the ligand-bound complexes and conformational changes induced by them using Desmond module of Schrodinger suite [78]. The protein–ligand complexes were simulated in an Optimized Potential for Liquid Simulations 3 (OPLS3) force field in a TIP4P solvated periodic box with 10 Å spacing. The solvation of the complexes was followed by neutralization, minimization for up to 2000 iterations. The minimized system was heated up to 300 K, equilibrated and simulated for a time period ranging between 50–110 ns. Root mean square deviation (RMSD), hydrogen bonds analysis and conformational changes over the simulation trajectories of protein–ligand complexes were monitored using VMD version 1.9.4 [85]. Images for this publication were generated using Pymol molecular graphics system [86]. The protein–protein binding energy in the presence and absence of inhibitors were calculated using PRODIGY webserver [84].

### 4.5. Immunostaining and Cell Congregation Analyses

Immunostaining: 25 × 10^3^ cells per well were seeded on glass coverslips placed in 12-well cell culture plates. After 24 h of treatment with the TEG derivatives, control or treated cells were fixed in methanol:acetone (1:1). Cells were permeabilized with 0.2% Tween-20 in phosphate-buffered saline (PBST), washed with phosphate-buffered saline (PBS) and blocked with 2% bovine serum albumin protein dissolved in PBST. Fixed cells were incubated with primary antibodies (as indicated) overnight, washed with PBS-PBST-PBS (5 min each), incubated with either Alexa-Fluor 488 goat anti-mouse IgG (Life Technologies, Carlsbad, CA, USA, A11029), Alexa-Fluor 594 rabbit anti-goat IgG (Life Technologies, Carlsbad, CA, USA, A11078) or Alexa-Fluor 594 goat anti-rabbit IgG (Life Technologies, Carlsbad, CA, USA, A11037) depending on the source of the primary antibodies, for 2 h, washed with PBS-PBST-PBS (5 min each), incubated with Hoechst 33342 stain (Invitrogen^®^, H3570) for 10 min, washed with PBST-PBS-ultrapure water (5 min each) and mounted on glass slides. The cells were then visualized for immunofluorescence under a microscope at ×400 magnification. Protein expression was quantified using ImageJ software (NIH, 1.52a) and plotted as a percentage using Microsoft™ Office© 2016.

Cell congregation analyses: Cells fixed on coverslips and stained with anti-E-cadherin primary antibody were selected and analyzed. Numbers of cells from 3 random fields were counted and investigated on the basis of viability, distance to the nearest neighbor (=congregation) and E-cadherin intensity using ImageJ software (NIH, Bathesda, MD, USA, 1.52a) and plotted as a percentage using Microsoft™ Office© 2016.

### 4.6. Western Blotting

50 × 10^4^ cells per well were seeded in 6-well plates and allowed to settle overnight, followed by the treatment with varying doses of the TEG derivatives. Control and treated cells were harvested and washed with PBS (×2) and lysed in RIPA buffer (Thermo Fisher Scientific, Waltham, MA, USA, 89900) containing complete protease inhibitor cocktail (Roche, Basel, Switzerland, 4693159001) on ice for 45 min. Mortalin-over-expressing and -compromised cells were prepared as described earlier [87,88]. Lysates were separated on an SDS-polyacrylamide gel using Mini-Protean^®^ Tetra cell equipment (Bio-Rad, Hercules, CA, USA) and subjected to Western blotting using protein-specific antibodies as indicated and horseradish peroxidase-conjugated secondary HRP antibody (Thermo Fisher Scientific, Waltham, MA, USA, 31430, 31402 or 31460). Blots were developed using chemiluminescence solution (GE Healthcare, Buckinghamshire, UK) and visualized using a Lumino Image Analyzer (LAS 3000-mini; Fuji Film, Tokyo, Japan). Band intensity was quantified using ImageJ software (NIH, Bathesda, MD, USA, 1.52a) and plotted as a percentage using Microsoft™ Office© 2016.

### 4.7. RT-PCR

50 × 10^4^ cells per well were seeded in a 6-well plate, allowed to settle overnight and treated with varying doses of the TEG derivatives. Control and treated cells were harvested and washed with PBS (×2) and lysed with TRIzol™ (Ambion^®^, Foster City, CA, USA, 15596018) at room temperature for 5 min, segregated in chloroform (Wako, Tokyo, Japan, 038-02606) at room temperature for 5 min, centrifuged at 12,000 rpm for 15 min and supernatant separated, washed in isopropanol (Wako, Osaka, Japan, 166-04836) at room temperature for 10 min, centrifuged at 12,000 rpm for 15 min and pellet-washed in 70% ice-cold ethanol and centrifuged at 8000 rpm for 5 min twice, followed by air-drying and resuspension in nuclease-free water to extract pure RNA. The concentration and quality of RNA were evaluated by a spectrophotometer (ND-1000, Nanodrops^®^, Wilmington, NC, USA). cDNA was prepared using a reverse transcription kit (Qiagen, Hilden, Germany, 205313) following the manufacturer’s instructions; purified mRNA samples were incubated with gDNA wipeout buffer to eliminate genomic DNA contaminants. The master mix for amplification was prepared by mixing 1 µL cDNA with 0.1 µL ExTaq (Takara, Kusatsu, Shiga, Japan, RR001), 2 µL 10× TAQ buffer, 2 µL dNTP-mix, 1 µL each of forward and reverse primers, in 12.9 µL nuclease-free water and amplified using “denaturation = 95 °C, 10 min → amplification = 95 °C, 60 s–x °C, 1 min–72 °C, y s (33 cycles) → annealing = 72 °C, 10 min → 4 °C” protocol. Primers for VEGF (F = agggcagaatcatcacgaagt/R = agggtctcgattggatggca, x = 64, y = 30), GAPDH (F = tggaaatcccatcaccatct/R = ttcacacccatgacgaacat, x = 61, y = 45), MMP-2 (F = tacaggatcattggctacacacc/R = ggtcacatcgctccagact, x = 64, y = 30), MMP-7 (F = gagtgagctacagtgggaaca/R = ctatgacgcgggagtttaacat, x = 61, y = 30), MMP-9 (F = tgtaccgctatggttacactcg/R = ggcagggacagttgcttct, x = 61, y = 30), Mortalin (F = agctggaatggccttagtcat/R = caggagttggtagtacccaaatc, x = 61, y = 45), E-cadherin (F = cgggaatgcagttgaggatc/R = aggatggtgtaagcgatggc, x = 64, y = 30) were used in the assay. The amplified products were separated on a 1% agarose gel containing 0.0625 µg/mL ethidium bromide (Invitrogen^®^, Carlsbad, CA, USA, 15585-011) and acquired using a Lumino Image Analyzer (LAS3000-mini; Fuji Film, Tokyo, Japan) equipped with a CCD (charge-coupled device) camera. Band intensity was quantified using ImageJ software (NIH, Bathesda, MD, USA, 1.52a) and plotted as a percentage using Microsoft™ Office© 2016.

### 4.8. VEGF Analyses

ELISA: 25 × 10^4^ cells per well were seeded in 6-well plates and allowed to settle overnight, followed by the treatment with varying doses of the TEG derivatives. Control and treated cells were harvested, washed with PBS (×2) and lysed in RIPA buffer (Thermo Fisher Scientific, Waltham, MA, USA, 89900) containing complete protease inhibitor cocktail (Roche, Basel, Switzerland, 4693159001) on ice for 45 min. Cell supernatant with secreted proteins (75 µg in 100 µL) was incubated in 96-well plate (Corning, NY, USA, 3632) pre-coated with anti-VEGF polyclonal antibody (100 ng/mL) in 100 µL coating and diluent buffers (DB) (BioLegend, San Diego, CA, USA, 421701 and 421203, respectively) at room temperature overnight. Wells were washed with 200 µL 0.05% Tween 20 in PBS three times for 1 min each and reloaded with an anti-VEGF antibody (100 ng/mL) in 100 µL DB. The plate was incubated at room temperature overnight followed by three washings of 1 min each and incubated with secondary horseradish peroxidase antibody (100 ng/mL) (Thermo Fisher Scientific, Waltham, MA, USA, 31430) in 100 µL DB at room temperature for 3 h. The plate was washed three times for 1 min each followed by incubation with 100 µL 3,3′,5,5′-tetramethylbenzidine substrate (BioLegend San Diego, CA, USA, 421101) for 30 min. One hundred microliters of Stop Solution (BioLegend, San Diego, CA, USA, 423001) was added, and optical density was measured at 450 nm using a spectrophotometer. Differences in VEGF absorbance at 450 nm wavelength were then expressed and plotted in percentage taking control as 100% using Microsoft™ Office© 2016.

Immunoprecipitation: Culture medium supernatant of the treated Panc-1 cells from VEGF ELISA was taken and incubated with Dyna beads^®^ Protein A (750 µL containing 750 g protein in each sample) conjugated with 0.25 µL rabbit anti-VEGF polyclonal antibody for overnight at 4 °C. The immunocomplexes were washed three times with PBS containing 0.2% Triton-X100 (Wako, Osaka, Japan, 162-24755) and eluted in denaturing SDS loading buffer (20 μL) by heating at 99 °C for 5 min. They were then separated on 8% SDS-polyacrylamide gel using Mini-Protean^®^ Tetra cell equipment (Bio-Rad, Hercules, CA, USA), along with controls (isotype and beads) and cell lysate (10 µg), and subjected to Western blotting using rabbit anti-VEGF antibody and anti-rabbit horseradish peroxidase-conjugated secondary HRP antibody (Thermo Fisher Scientific, Waltham, MA, USA, 31430). 

### 4.9. Statistical Analyses

All the quantifications were performed using ImageJ software (NIH, Bathesda, MD, USA, 1.52a), and calculations and plots were created using Microsoft™ Office© 2016. Statistical significance was calculated using mean, N (number) and SD (standard deviation) by unpaired t-test of Microsoft™ Excel© 2016 from at least three independent experiments and shown as * *p* < 0.05, ** *p* < 0.01, *** *p* < 0.001, ns = not significant.

## Figures and Tables

**Table 1:** Binding energies of TD-10 and TD-11 with MMP-2, MMP-7 and MMP-9 protein.

Parameter	MMP-2		MMP-9		MMP-7	
	TD-10	TD-11	TD-10	TD-11	TD-10	TD-11
**Docking Score (kCal/mol)**	−5.80	−7.78	−5.70	−5.81	−4.98	−6.15
**MM-GBSA Binding energy (kCal/mol)**	−63.55	−74.81	−58.44	−45.78	−15.86	−28.97

## Supplementary Materials

Supplementary materials can be found at https://www.mdpi.com/1422-0067/21/15/5463/s1. Figure S1. MTT-based dose-titration assay to determine the non-toxic (IC_10/50_) dose of TD-10 and TD-11 in Panc-1,MDA-MB-231, HeLa, DLD-1, T.T. and HSC3 cells; Figure S2. Wound scratch migration assay to show a delay in migration by the non-toxic doses of TD-10 and TD-11 in MDA-MB-231 and HeLa cells; Figure S3. Western blotting results to show the effects of TD-10 and TD-11 treatment on the protein expression of VEGF and MMPs in MDA-MB-231 and HeLa cells; Figure S4. Immunostaining results to show the effects of TD-10 and TD-11 treatment on the protein expression of VEGF and MMPs in MDA-MB-231 and HeLa cells; Figure S5. RT-PCR results to show the effects of TD-10 and TD-11 treatment on the mRNA expression of VEGF and MMPs in MDA-MB-231 and HeLa cells; Table S1. Binding energy of TD-10 and TD-11 with vimentin, mortalin, p53 and hnRNP-K.

## Abbreviations

EMT	Epithelial-to-mesenchymal transformation
MMP	Matrix metalloprotease
TD-10	Triethylene glycol dimethacrylate-10
TD-11	Triethylene glycol dimethacrylate-11
TEG	Triethylene glycol
VEGF	Vascular endothelial growth factor
