# Supplementary figures and images for: Bioinformatics and Molecular Insights to Anti-Metastasis Activity of Triethylene Glycol Derivatives
Source: Int J Mol Sci. 2020 Jul 30;21(15):5463. doi: 10.3390/ijms21155463 (PMC7432423; doi:10.3390/ijms21155463)

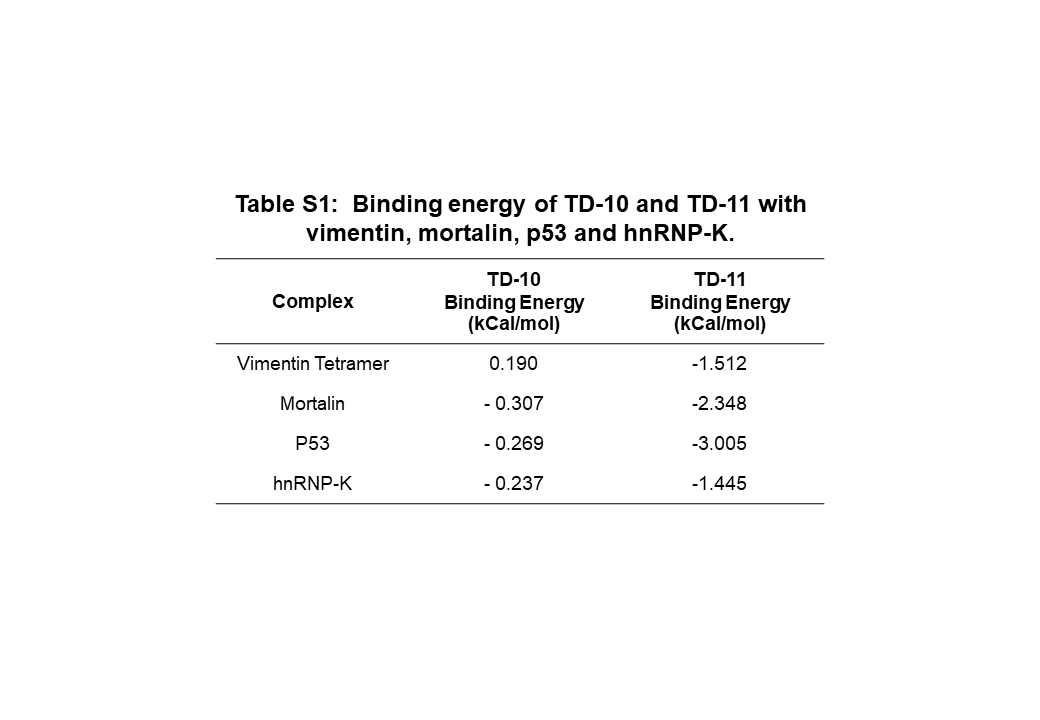

Supplement: Supplementary file 1 [file ijms-21-05463-s001.zip › Malik et al IJMS2020 Suppl Files/Table S1.TIF]

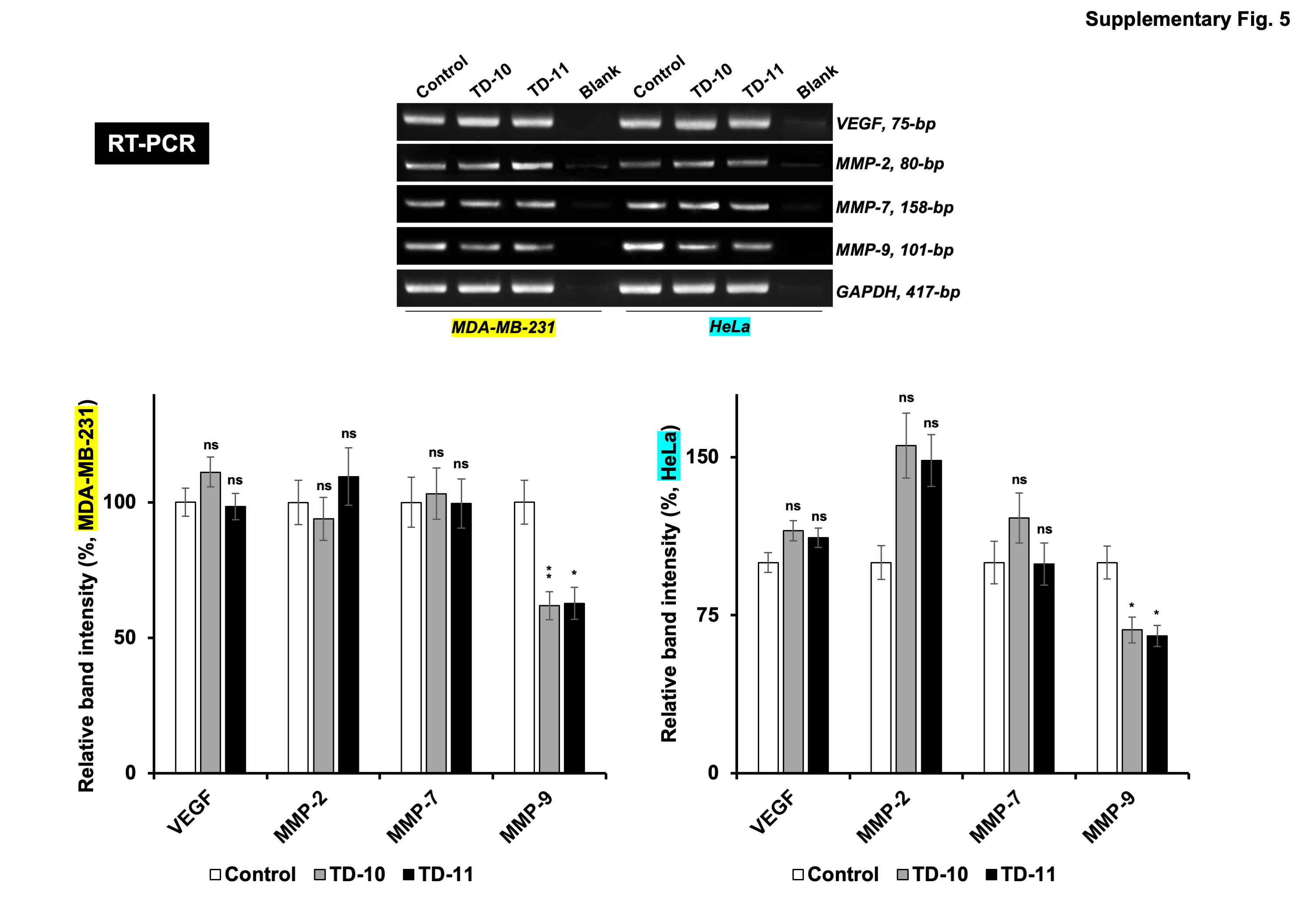

Supplement: Supplementary file 1 [file ijms-21-05463-s001.zip › Malik et al IJMS2020 Suppl Files/Figure S5.tiff]

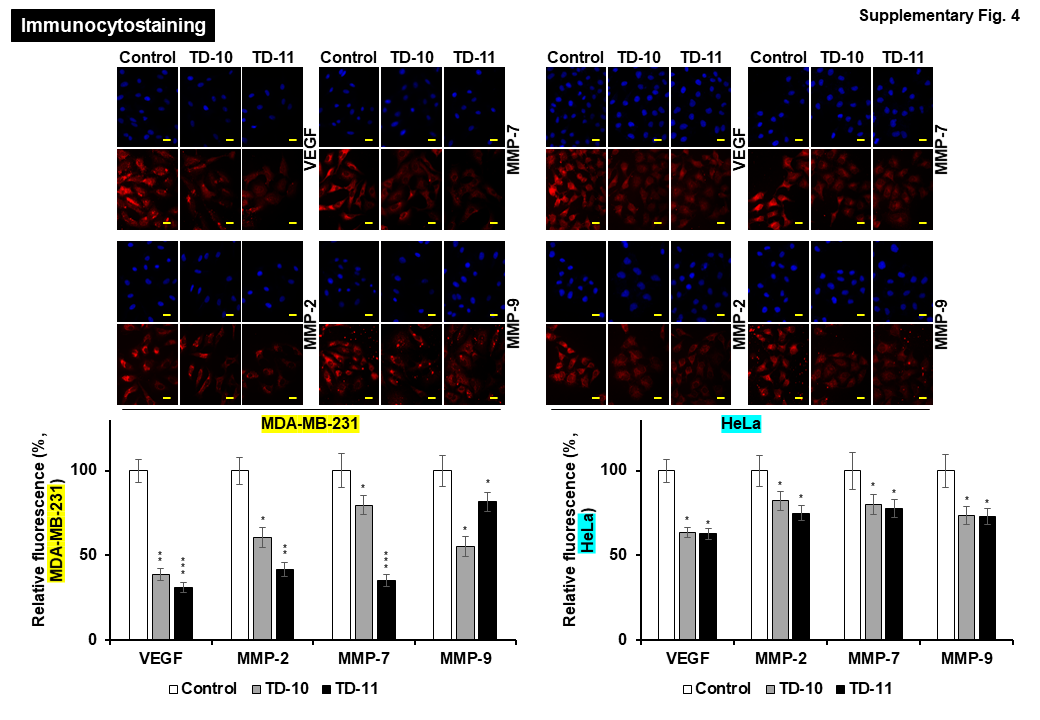

Supplement: Supplementary file 1 [file ijms-21-05463-s001.zip › Malik et al IJMS2020 Suppl Files/Figure S4.TIF]

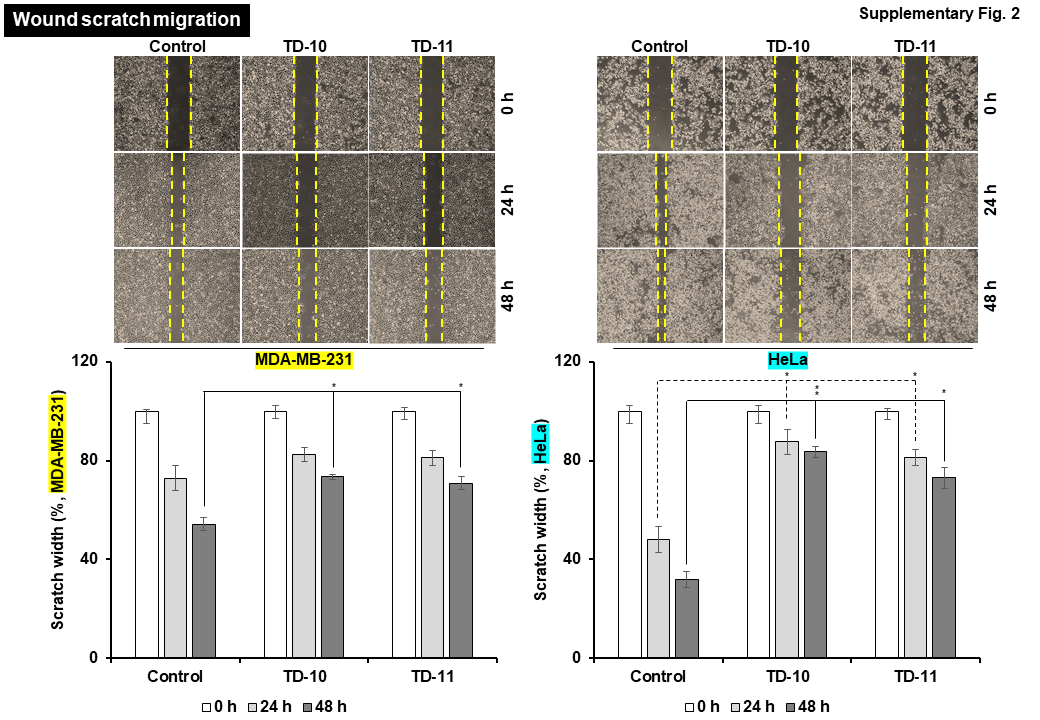

Supplement: Supplementary file 1 [file ijms-21-05463-s001.zip › Malik et al IJMS2020 Suppl Files/Figure S2.TIF]

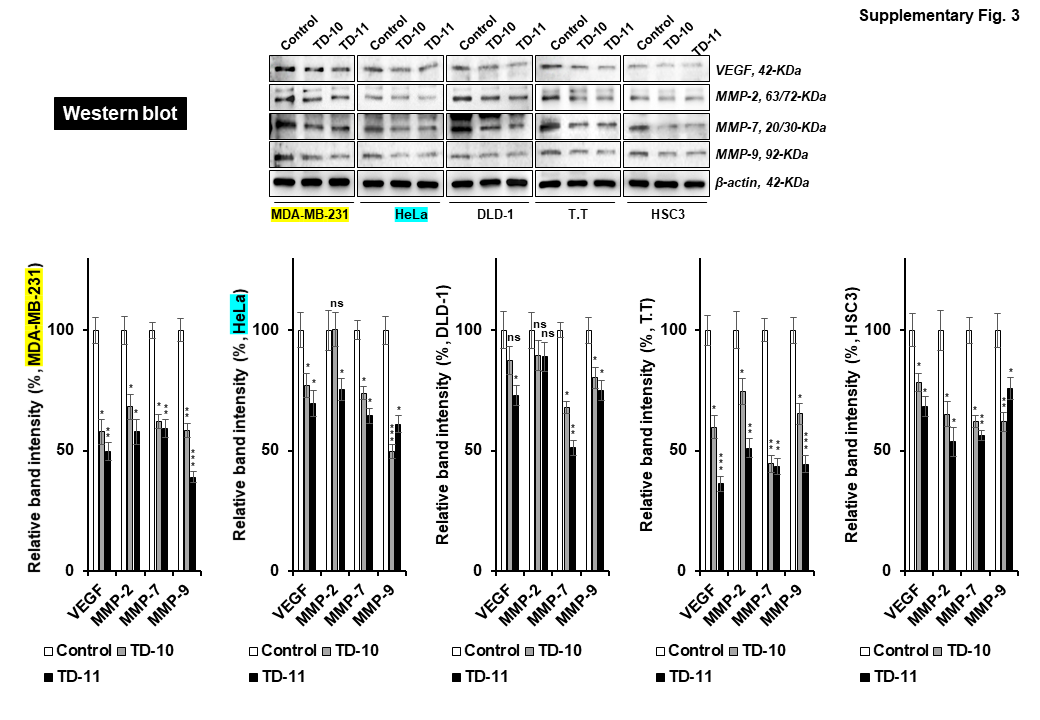

Supplement: Supplementary file 1 [file ijms-21-05463-s001.zip › Malik et al IJMS2020 Suppl Files/Figure S3.TIF]

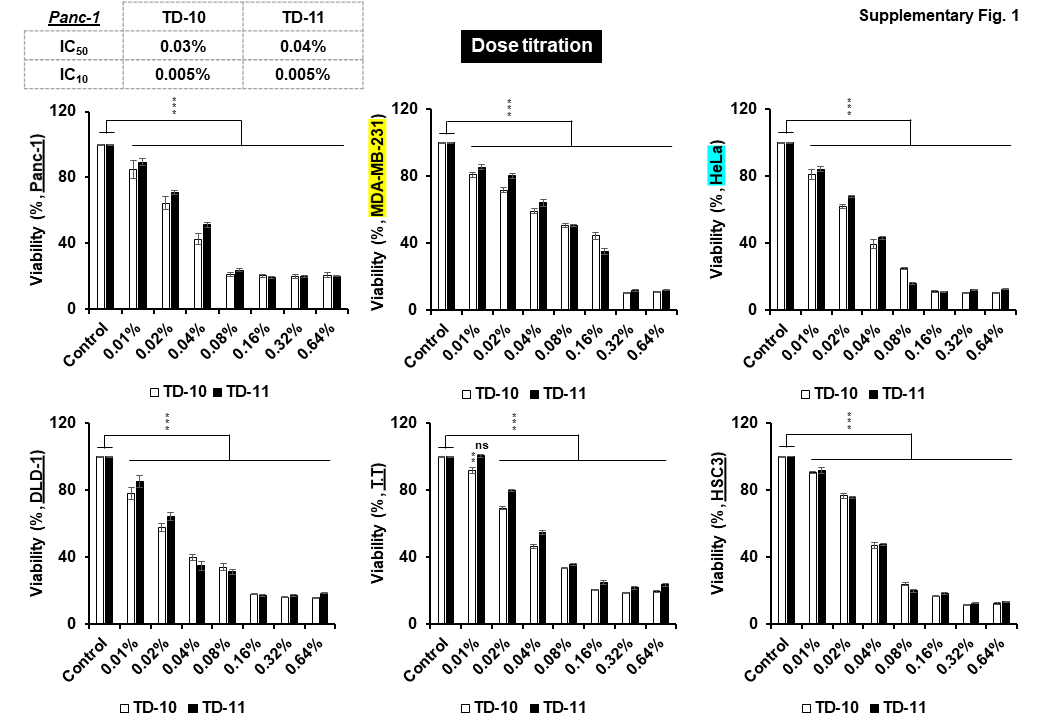

Supplement: Supplementary file 1 [file ijms-21-05463-s001.zip › Malik et al IJMS2020 Suppl Files/Figure S1.TIF]
